# (*E*)-*N*′-(1,3-Benzodioxol-5-ylmethyl­ene)nicotinohydrazide monohydrate

**DOI:** 10.1107/S1600536809034503

**Published:** 2009-09-05

**Authors:** Feng-Yu Bao, Hai-Yan Zhang, Ying-Xia Zhou, Su Hui

**Affiliations:** a Department of Applied Chemistry, College of Sciences, Henan Agricultural University, Zhengzhou 450002, People’s Republic of China

## Abstract

In the title compound, C_14_H_11_N_3_O_3_·H_2_O, the planar [maximum deviation 0.135 (1) Å] 1,3-benzodioxole ring system is oriented at a dihedral angle of 13.93 (7)° with respect to the pyridine ring. Extensive inter­molecular N—H⋯O, O—H⋯O, O—H⋯N and weak C—H⋯O hydrogen bonding is present in the crystal structure.

## Related literature

For applications of Schiff base compounds, see: Kahwa *et al.* (1986 ▶); Santos *et al.* (2001 ▶).
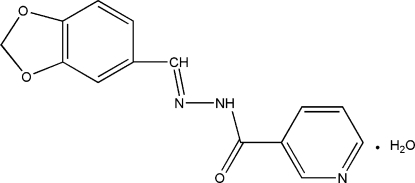


## Experimental

### 

#### Crystal data

C_14_H_11_N_3_O_3_·H_2_O
*M* *_r_* = 287.28
Monoclinic, 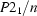

*a* = 8.6414 (2) Å
*b* = 12.0874 (2) Å
*c* = 13.4464 (3) Å
β = 103.161 (1)°
*V* = 1367.61 (5) Å^3^
*Z* = 4
Mo *K*α radiation
μ = 0.11 mm^−1^
*T* = 293 K
0.26 × 0.21 × 0.17 mm

#### Data collection

Bruker SMART CCD area-detector diffractometer
Absorption correction: multi-scan (*SADABS*; Bruker, 1998 ▶) *T*_min_ = 0.974, *T*_max_ = 0.982
19494 measured reflections
2691 independent reflections
1839 reflections with *I* > 2σ(*I*)
*R*_int_ = 0.044

#### Refinement

*R*[*F* ^2^ > 2σ(*F* ^2^)] = 0.041
*wR*(*F* ^2^) = 0.112
*S* = 1.04
2691 reflections
199 parameters
3 restraints
H atoms treated by a mixture of independent and constrained refinement
Δρ_max_ = 0.15 e Å^−3^
Δρ_min_ = −0.13 e Å^−3^

### 

Data collection: *SMART* (Bruker, 1998 ▶); cell refinement: *SAINT* (Bruker, 1998 ▶); data reduction: *SAINT*; program(s) used to solve structure: *SHELXTL* (Sheldrick, 2008 ▶); program(s) used to refine structure: *SHELXTL*; molecular graphics: *SHELXTL*; software used to prepare material for publication: *SHELXTL*

## Supplementary Material

Crystal structure: contains datablocks global, I. DOI: 10.1107/S1600536809034503/xu2599sup1.cif

Structure factors: contains datablocks I. DOI: 10.1107/S1600536809034503/xu2599Isup2.hkl

Additional supplementary materials:  crystallographic information; 3D view; checkCIF report

## Figures and Tables

**Table 1:** Hydrogen-bond geometry (Å, °)

*D*—H⋯*A*	*D*—H	H⋯*A*	*D*⋯*A*	*D*—H⋯*A*
N2—H2*A*⋯O4^i^	0.86	2.04	2.8819 (17)	164
O4—H4*A*⋯N3	0.848 (9)	1.980 (17)	2.825 (2)	174 (2)
O4—H4*B*⋯O3^ii^	0.839 (9)	2.106 (15)	2.9019 (19)	158.2 (16)
C3—H3*B*⋯O3^iii^	0.97	2.57	3.495 (2)	160
C8—H8*A*⋯O4^i^	0.93	2.51	3.312 (2)	144
C11—H11*A*⋯O4^i^	0.93	2.45	3.324 (2)	156

Symmetry codes: (i) 
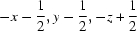
; (ii) 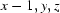; (iii) 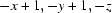.

**Fig. 1.**
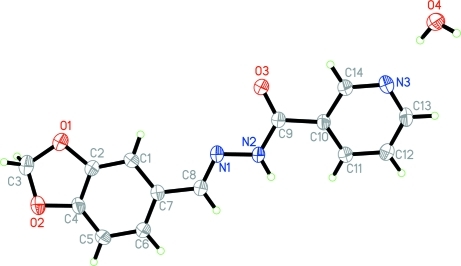
The molecular structure of the title compound. Displacement ellipsoids are drawn at the 30% probability level.

**Fig. 2.**
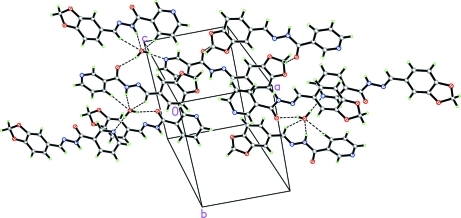
The unit cell packing diagram showing intermolecular hydrogen bonding as dashed lines.

## supplementary crystallographic information

### Comment

The chemistry of Schiff bases has attracted a great deal of interest in recent years. These compounds play an important role in the development of various proteins and enzymes (Kahwa *et al.*, 1986; Santos *et al.*, 2001). As part of our interest in the coordination chemistry of Schiff bases, we have synthesized the title compound and report here its crystal structure.

The title molecule crystallizes in the E conformation (Fig. 1), with the N2—N1—C8—C7 torsion angle of 179.60 (13)°. The dihedral angle between the 1,3-benzodioxole ring system and the pyridine ring is 13.93 (7)°. The extensive intermolecular classic N—H···O, O—H···O, O—H···N and weak C—H···O hydrogen bonding is present in the crystal structure (Table 1 and Fig. 2).

### Experimental

Nicotinohydrazide (1 mmol, 0.137 g) was dissolved in ethanol (15 ml). The solution was stirred at 351 K for several min, and then the 1,3-benzodioxole-5-carbaldehyde (1 mmol, 0.150 g) in ethanol (8 mm l) was added dropwise. The mixture was refluxed for 2 h. The solid product was isolated and recrystallized from methanol-water solution. Colourless single crystals of the title compound were obtained after 3 d.

### Refinement

H atoms of water molecule are located in a difference Fourier map and refined isotropically with O—H and H···H distances restrained to 0.85 (1) and 1.37 (2) A. Other H atoms were positioned geometrically and refined as riding with C—H = 0.93 (aromatic), 0.97 Å (methylene) and N—H = 0.86 Å, *U*_iso_(H) = 1.2*U*_eq_(C,*N*).

### Crystal data

**Table:**

C_14_H_11_N_3_O_3_·H_2_O	*F*(000) = 600
*M**_r_* = 287.28	*D*_x_ = 1.395 Mg m^−^^3^
Monoclinic, *P*2_1_/*n*	Mo *K*α radiation, λ = 0.71073 Å
Hall symbol: -P 2yn	Cell parameters from 2884 reflections
*a* = 8.6414 (2) Å	θ = 2.3–26.0°
*b* = 12.0874 (2) Å	µ = 0.11 mm^−^^1^
*c* = 13.4464 (3) Å	*T* = 293 K
β = 103.161 (1)°	Block, colourless
*V* = 1367.61 (5) Å^3^	0.26 × 0.21 × 0.17 mm
*Z* = 4	

### Data collection

**Table:**

Bruker SMART CCD area-detector diffractometer	2691 independent reflections
Radiation source: fine-focus sealed tube	1839 reflections with *I* > 2σ(*I*)
graphite	*R*_int_ = 0.044
ω scans	θ_max_ = 26.0°, θ_min_ = 2.3°
Absorption correction: multi-scan (*SADABS*; Bruker, 1998)	*h* = −10→10
*T*_min_ = 0.974, *T*_max_ = 0.982	*k* = −14→14
19494 measured reflections	*l* = −16→16

### Refinement

**Table:**

Refinement on *F*^2^	Secondary atom site location: difference Fourier map
Least-squares matrix: full	Hydrogen site location: inferred from neighbouring sites
*R*[*F*^2^ > 2σ(*F*^2^)] = 0.041	H atoms treated by a mixture of independent and constrained refinement
*wR*(*F*^2^) = 0.112	*w* = 1/[σ^2^(*F*_o_^2^) + (0.0582*P*)^2^ + 0.0682*P*] where *P* = (*F*_o_^2^ + 2*F*_c_^2^)/3
*S* = 1.04	(Δ/σ)_max_ = 0.010
2691 reflections	Δρ_max_ = 0.15 e Å^−^^3^
199 parameters	Δρ_min_ = −0.13 e Å^−^^3^
3 restraints	Extinction correction: *SHELXTL* (Version 5.1; Sheldrick, 2008), Fc^*^=kFc[1+0.001xFc^2^λ^3^/sin(2θ)]^-1/4^
Primary atom site location: structure-invariant direct methods	Extinction coefficient: 0.0058 (14)

### Special details

**Table:**

Geometry. All e.s.d.'s (except the e.s.d. in the dihedral angle between two l.s. planes) are estimated using the full covariance matrix. The cell e.s.d.'s are taken into account individually in the estimation of e.s.d.'s in distances, angles and torsion angles; correlations between e.s.d.'s in cell parameters are only used when they are defined by crystal symmetry. An approximate (isotropic) treatment of cell e.s.d.'s is used for estimating e.s.d.'s involving l.s. planes.
Refinement. Refinement of *F*^2^ against ALL reflections. The weighted *R*-factor *wR* and goodness of fit *S* are based on *F*^2^, conventional *R*-factors *R* are based on *F*, with *F* set to zero for negative *F*^2^. The threshold expression of *F*^2^ > σ(*F*^2^) is used only for calculating *R*-factors(gt) *etc*. and is not relevant to the choice of reflections for refinement. *R*-factors based on *F*^2^ are statistically about twice as large as those based on *F*, and *R*- factors based on ALL data will be even larger.

### Fractional atomic coordinates and isotropic or equivalent isotropic displacement parameters (Å^2^)

**Table:**

	*x*	*y*	*z*	*U*_iso_*/*U*_eq_	
N1	0.25573 (14)	0.30595 (11)	0.16863 (10)	0.0528 (4)	
C7	0.46242 (17)	0.23623 (12)	0.09399 (11)	0.0469 (4)	
O3	0.08396 (13)	0.45982 (9)	0.23857 (10)	0.0702 (4)	
C10	−0.09502 (16)	0.32960 (12)	0.28168 (11)	0.0442 (4)	
O2	0.84690 (15)	0.27321 (11)	−0.03215 (11)	0.0807 (4)	
N2	0.12618 (14)	0.27993 (10)	0.20830 (10)	0.0516 (4)	
H2A	0.0972	0.2122	0.2116	0.062*	
C2	0.65230 (18)	0.34247 (13)	0.03595 (12)	0.0522 (4)	
N3	−0.33050 (15)	0.39773 (11)	0.32710 (11)	0.0596 (4)	
O1	0.73361 (16)	0.43293 (10)	0.01301 (11)	0.0842 (5)	
C6	0.53331 (19)	0.14169 (13)	0.06740 (13)	0.0544 (4)	
H6A	0.4908	0.0732	0.0781	0.065*	
C8	0.32492 (19)	0.22390 (13)	0.13835 (12)	0.0530 (4)	
H8A	0.2860	0.1532	0.1449	0.064*	
C11	−0.12747 (19)	0.22473 (13)	0.31162 (13)	0.0523 (4)	
H11A	−0.0606	0.1659	0.3059	0.063*	
C5	0.66608 (19)	0.14538 (13)	0.02518 (13)	0.0576 (5)	
H5A	0.7142	0.0814	0.0084	0.069*	
C13	−0.35719 (19)	0.29587 (15)	0.35623 (13)	0.0607 (5)	
H13A	−0.4466	0.2835	0.3822	0.073*	
C4	0.72117 (19)	0.24715 (14)	0.00991 (13)	0.0530 (4)	
C9	0.04620 (17)	0.36199 (12)	0.24153 (12)	0.0489 (4)	
C1	0.52308 (19)	0.34048 (12)	0.07778 (12)	0.0520 (4)	
H1B	0.4771	0.4053	0.0948	0.062*	
C12	−0.26006 (19)	0.20836 (14)	0.34996 (13)	0.0606 (5)	
H12A	−0.2834	0.1384	0.3714	0.073*	
C14	−0.20100 (18)	0.41282 (13)	0.29066 (12)	0.0523 (4)	
H14A	−0.1805	0.4837	0.2700	0.063*	
C3	0.8597 (2)	0.38998 (17)	−0.02752 (16)	0.0772 (6)	
H3B	0.8535	0.4197	−0.0953	0.093*	
H3C	0.9612	0.4112	0.0157	0.093*	
O4	−0.59280 (17)	0.54330 (10)	0.28071 (14)	0.0854 (5)	
H4A	−0.5119 (16)	0.5021 (15)	0.2985 (18)	0.112 (8)*	
H4B	−0.6745 (16)	0.5040 (15)	0.2633 (16)	0.102 (8)*	

### Atomic displacement parameters (Å^2^)

**Table:**

	*U*^11^	*U*^22^	*U*^33^	*U*^12^	*U*^13^	*U*^23^
N1	0.0450 (7)	0.0537 (8)	0.0663 (9)	−0.0044 (6)	0.0260 (7)	−0.0009 (7)
C7	0.0436 (8)	0.0483 (9)	0.0525 (9)	−0.0015 (7)	0.0187 (7)	−0.0012 (7)
O3	0.0679 (8)	0.0461 (7)	0.1111 (10)	−0.0071 (5)	0.0505 (7)	−0.0048 (6)
C10	0.0375 (8)	0.0476 (9)	0.0491 (9)	−0.0033 (6)	0.0135 (7)	−0.0031 (7)
O2	0.0748 (9)	0.0758 (9)	0.1108 (11)	−0.0141 (7)	0.0614 (8)	−0.0153 (7)
N2	0.0440 (7)	0.0454 (7)	0.0729 (9)	−0.0049 (6)	0.0294 (7)	−0.0021 (6)
C2	0.0550 (10)	0.0463 (9)	0.0600 (10)	−0.0091 (7)	0.0234 (8)	−0.0017 (7)
N3	0.0461 (8)	0.0603 (9)	0.0782 (10)	0.0029 (7)	0.0260 (7)	−0.0028 (7)
O1	0.0930 (10)	0.0566 (8)	0.1237 (11)	−0.0162 (7)	0.0675 (9)	−0.0050 (7)
C6	0.0545 (10)	0.0448 (9)	0.0698 (11)	−0.0030 (7)	0.0262 (8)	−0.0015 (8)
C8	0.0500 (9)	0.0483 (9)	0.0663 (11)	−0.0057 (7)	0.0249 (8)	−0.0030 (8)
C11	0.0499 (9)	0.0474 (9)	0.0645 (10)	0.0006 (7)	0.0235 (8)	0.0002 (8)
C5	0.0563 (10)	0.0476 (10)	0.0754 (12)	0.0023 (7)	0.0286 (9)	−0.0088 (8)
C13	0.0483 (10)	0.0688 (11)	0.0733 (12)	−0.0067 (8)	0.0312 (9)	−0.0040 (9)
C4	0.0455 (9)	0.0601 (10)	0.0597 (10)	−0.0035 (7)	0.0252 (8)	−0.0067 (8)
C9	0.0448 (9)	0.0466 (9)	0.0593 (10)	−0.0027 (7)	0.0200 (8)	0.0003 (7)
C1	0.0540 (9)	0.0454 (9)	0.0621 (10)	0.0009 (7)	0.0245 (8)	−0.0062 (7)
C12	0.0624 (11)	0.0522 (10)	0.0754 (12)	−0.0086 (8)	0.0329 (10)	0.0020 (8)
C14	0.0472 (9)	0.0467 (9)	0.0672 (10)	0.0015 (7)	0.0216 (8)	0.0019 (8)
C3	0.0742 (13)	0.0751 (13)	0.0953 (15)	−0.0113 (11)	0.0461 (11)	0.0042 (11)
O4	0.0518 (8)	0.0474 (7)	0.1622 (15)	0.0012 (6)	0.0353 (9)	0.0012 (8)

### Geometric parameters (Å, °)

**Table:**

N1—C8	1.2719 (19)	O1—C3	1.423 (2)
N1—N2	1.3815 (16)	C6—C5	1.392 (2)
C7—C6	1.382 (2)	C6—H6A	0.9300
C7—C1	1.401 (2)	C8—H8A	0.9300
C7—C8	1.453 (2)	C11—C12	1.374 (2)
O3—C9	1.2299 (17)	C11—H11A	0.9300
C10—C11	1.378 (2)	C5—C4	1.351 (2)
C10—C14	1.384 (2)	C5—H5A	0.9300
C10—C9	1.4946 (19)	C13—C12	1.365 (2)
O2—C4	1.3709 (18)	C13—H13A	0.9300
O2—C3	1.416 (2)	C1—H1B	0.9300
N2—C9	1.3423 (18)	C12—H12A	0.9300
N2—H2A	0.8600	C14—H14A	0.9300
C2—C1	1.360 (2)	C3—H3B	0.9700
C2—O1	1.3723 (18)	C3—H3C	0.9700
C2—C4	1.378 (2)	O4—H4A	0.848 (9)
N3—C13	1.328 (2)	O4—H4B	0.839 (9)
N3—C14	1.3324 (19)		
			
C8—N1—N2	115.31 (13)	C6—C5—H5A	121.9
C6—C7—C1	120.01 (14)	N3—C13—C12	123.35 (15)
C6—C7—C8	118.26 (13)	N3—C13—H13A	118.3
C1—C7—C8	121.73 (14)	C12—C13—H13A	118.3
C11—C10—C14	117.41 (13)	C5—C4—O2	127.70 (15)
C11—C10—C9	125.71 (14)	C5—C4—C2	122.33 (14)
C14—C10—C9	116.88 (13)	O2—C4—C2	109.96 (14)
C4—O2—C3	105.81 (12)	O3—C9—N2	122.63 (13)
C9—N2—N1	118.97 (12)	O3—C9—C10	120.53 (13)
C9—N2—H2A	120.5	N2—C9—C10	116.83 (13)
N1—N2—H2A	120.5	C2—C1—C7	116.86 (14)
C1—C2—O1	128.15 (14)	C2—C1—H1B	121.6
C1—C2—C4	122.21 (14)	C7—C1—H1B	121.6
O1—C2—C4	109.64 (13)	C13—C12—C11	119.27 (16)
C13—N3—C14	116.88 (14)	C13—C12—H12A	120.4
C2—O1—C3	105.75 (13)	C11—C12—H12A	120.4
C7—C6—C5	122.29 (14)	N3—C14—C10	124.09 (15)
C7—C6—H6A	118.9	N3—C14—H14A	118.0
C5—C6—H6A	118.9	C10—C14—H14A	118.0
N1—C8—C7	122.63 (15)	O2—C3—O1	108.74 (13)
N1—C8—H8A	118.7	O2—C3—H3B	109.9
C7—C8—H8A	118.7	O1—C3—H3B	109.9
C12—C11—C10	118.99 (15)	O2—C3—H3C	109.9
C12—C11—H11A	120.5	O1—C3—H3C	109.9
C10—C11—H11A	120.5	H3B—C3—H3C	108.3
C4—C5—C6	116.29 (14)	H4A—O4—H4B	109.5 (18)
C4—C5—H5A	121.9		

### Hydrogen-bond geometry (Å, °)

**Table:**

*D*—H···*A*	*D*—H	H···*A*	*D*···*A*	*D*—H···*A*
N2—H2A···O4^i^	0.86	2.04	2.8819 (17)	164
O4—H4A···N3	0.85 (1)	1.98 (2)	2.825 (2)	174 (2)
O4—H4B···O3^ii^	0.84 (1)	2.11 (2)	2.9019 (19)	158 (2)
C3—H3B···O3^iii^	0.97	2.57	3.495 (2)	160
C8—H8A···O4^i^	0.93	2.51	3.312 (2)	144
C11—H11A···O4^i^	0.93	2.45	3.324 (2)	156

Symmetry codes: (i) −*x*−1/2, *y*−1/2, −*z*+1/2; (ii) *x*−1, *y*, *z*; (iii) −*x*+1, −*y*+1, −*z*.

## References

[bb1] Bruker (1998). *SMART*, *SAINT* and *SADABS* Bruker AXS Inc., Madison, Wisconsin, USA.

[bb2] Kahwa, I. A., Selbin, I., Hsieh, T. C. Y. & Laine, R. A. (1986). * Inorg. Chim. Acta*, **118**, 179–185.

[bb3] Santos, M. L. P., Bagatin, I. A., Pereira, E. M. & Ferreira, A. M. D. C. (2001). *J. Chem. Soc. Dalton Trans.* pp. 838–844.

[bb4] Sheldrick, G. M. (2008). *Acta Cryst.* A**64**, 112–122. 10.1107/S0108767307043930 18156677

